# DNA Methylation Targets Influenced by Bisphenol A and/or Genistein Are Associated with Survival Outcomes in Breast Cancer Patients

**DOI:** 10.3390/genes8050144

**Published:** 2017-05-15

**Authors:** Rohit R. Jadhav, Julia Santucci-Pereira, Yao V. Wang, Joseph Liu, Theresa D. Nguyen, Jun Wang, Sarah Jenkins, Jose Russo, Tim H.-M. Huang, Victor X. Jin, Coral A. Lamartiniere

**Affiliations:** 1 Department of Molecular Medicine, University of Texas Health Science Center at San Antonio, 8403 Floyd Curl, San Antonio, TX 78229, USA; Jadhav@livemail.uthscsa.edu (R.R.J.); YaoWangPRC@hotmail.com (Y.V.W.); jliuj6@uthscsa.edu (J.L.); huangt3@uthscsa.edu (T.H.-M.H.); 2 Fox Chase Cancer Center, Temple University Health System, 333 Cottman Ave, Philadelphia, PA 19111, USA; Julia.Pereira@fccc.edu (J.S.-P.); theresa.diepnguyen@gmail.com (T.D.N.); Jose.Russo@fccc.edu (J.R.); 3 Department of Pharmacology and Toxicology University of Alabama at Birmingham, 1670 University Boulevard, Birmingham, AL 35294, USA; feijun@uab.edu (J.W.); jenkinss@uab.edu (S.J.)

**Keywords:** DNA-methylation, Bisphenol A, genistein

## Abstract

Early postnatal exposures to Bisphenol A (BPA) and genistein (GEN) have been reported to predispose for and against mammary cancer, respectively, in adult rats. Since the changes in cancer susceptibility occurs in the absence of the original chemical exposure, we have investigated the potential of epigenetics to account for these changes. DNA methylation studies reveal that prepubertal BPA exposure alters signaling pathways that contribute to carcinogenesis. Prepubertal exposure to GEN and BPA + GEN revealed pathways involved in maintenance of cellular function, indicating that the presence of GEN either reduces or counters some of the alterations caused by the carcinogenic properties of BPA. We subsequently evaluated the potential of epigenetic changes in the rat mammary tissues to predict survival in breast cancer patients via the Cancer Genomic Atlas (TCGA). We identified 12 genes that showed strong predictive values for long-term survival in estrogen receptor positive patients. Importantly, two genes associated with improved long term survival, *HPSE* and *RPS9*, were identified to be hypomethylated in mammary glands of rats exposed prepuberally to GEN or to GEN + BPA respectively, reinforcing the suggested cancer suppressive properties of GEN.

## 1. Introduction

Studies demonstrate that exposures to endogenous hormones and environmental chemicals during early development affect breast cancer incidence [1,2,3]. However, the underlying mechanism(s) of how early life exposure to hormonally-active chemicals can cause long-term alterations to a target tissue remains elusive. Two chemicals that exert permanent alterations to the biochemical blue-print of the mammary tissue and result in differential susceptibility for cancer are the endocrine disruptor Bisphenol A (BPA) and the phytoestrogen genistein (GEN) [1,2,3]. BPA is a high-volume chemical used as a monomer to manufacture polycarbonate plastics and the epoxy resins that line most canned foods and beverages. It has also been found in children’s toys, dental sealants, and cash register receipts. It is produced worldwide, with an estimated 7% annual growth due to manufacturing demand. Time, heat, and acidic or basic conditions accelerate hydrolysis of the ester bond linking BPA monomers, leading to the release of BPA into foods and liquids, thus making human environmental exposure inevitable and ubiquitous [4]. The United States Center for Disease Control has reported detectable levels of BPA in urine samples from 92.6% of more than 2500 participants in the cross-sectional National Health and Nutrition Examination Survey study [5]. BPA has been detected in amniotic fluid, maternal and fetal plasma, placental tissue of pregnant women, and in breast milk of lactating mothers [6,7]. Laboratory studies report BPA enhancing cell proliferation and decreasing apoptosis [3,8,9] and in vivo studies demonstrate prepubertal exposure to BPA results in adult rats being more susceptible for chemically-induced mammary cancer [2,10,11]. In France, all food containers containing BPA are banned (French rule n° 2012-1442). In addition, the European Union has prohibited BPA in infant feeding bottles since 2011.

On the other hand, epidemiological reports suggest individuals consuming a diet high in soy have a reduced risk of developing breast cancer [12,13,14,15,16,17], and in vivo studies demonstrate prepubertal GEN exposure suppresses chemically-induced mammary cancer in rats [18,19,20,21,22]. GEN is a natural component of soy-based products including tofu, soymilk, and soy infant formula. It is also found in soy-fortified foods and some over-the-counter dietary supplements. Dietary exposure to GEN can reach up to 1 mg/kg body weight (BW) in adults and up to 10-fold higher levels in infants fed milk formulas containing soy [23]. The level of GEN exposure in Asian populations consuming a soy-rich diet has been reported to range from ≈1 to 30 mg/d, or ~0.02–0.55 mg/kg BW × d, and considerably less in Western populations [24].

Although both BPA and GEN possess weak estrogenic properties, these chemicals influence the mammary gland via multiple mechanisms, including effects on signaling pathways, cell proliferation, rate of apoptosis, and DNA methylation to result in long term alterations [1,2,3,8,9,25,26,27,28,29,30,31,32,33]. Hsu et al. have shown that prepubertal BPA exposure results in epigenetic changes in the adult rat mammary gland [28]. Furthermore, it has been reported that BPA is able to increase the likelihood of developing precancerous lesions in adult tissues through altering DNA methylation at key cell signaling genes in humans [26], mice [25,32], and rats [27,29]. Meanwhile, studies have shown that GEN can prevent tumorigenesis in many cancers through targeting and epigenetically regulating gene expression, including DNA methylation, histone modifications, and non-coding RNAs [30]. Although effects observed in our previous study found prepubertal exposure to BPA and GEN, alone or in combination, can be attributed to a “programming” effect on the mammary proteome [33,34], whether these chemical exposures would profoundly affect the epigenetic landscape remains to be elucidated. To further explore such effects, we have performed a genome-wide methyl-binding domain sequencing (MBDCap-seq), on mammary glands of 100 day-old rats exposed prepubertally only to BPA and GEN, alone or in combination. Also, we performed network analysis for the genes identified as being differentially methylated and assessed the potential of these genes to predict long-term survival in breast cancer patients.

## 2. Materials and Methods

### 2.1. BPA and GEN Exposures and Mammary Gland Procurement

Animal care and treatments were performed according to established guidelines, and protocols were approved by the University of Alabama at Birmingham Animal Care Committee (University of Alabama at Birmingham Animal Project Number 111109280, approved 7 October 2011). Seven week old female Sprague-Dawley rats were purchased from Charles River Laboratories (Wilmington, MA, USA). Animals were housed in a temperature controlled facility with a 12-h light:dark cycle. These animals were bred with proven Sprague-Dawley studs, fed phytoestrogen-free AIN-76A diet (Harlan Teklad, Madison, WI, USA), housed in polypropylene cages, and provided with water via glass bottles (all polycarbonate/BPA free). On the day of birth (designated as postnatal day (PND) zero), offspring were sexed and litters were culled to 10 offspring per lactating dam. From PND2 through PND20 only, the offspring nursed via the lactating dams as listed in Table 1. Animals were weaned at PND21 and provided AIN-76A diet and water without further treatment/exposure.

GEN was provided by DSM Nutritional Products (Basel, Switzerland). BPA, sesame oil, and all other chemicals were from Sigma Chemical Co. (St. Louis, MO, USA). At PND100, female offspring were killed in the estrous phase. Individual animals (one) from five liters of each treatment were utilized. The fourth abdominal mammary glands were rapidly dissected from live ketamine/xylazine anesthetized animals (to minimize proteolysis), snap-frozen in liquid nitrogen, and stored at −80 °C for later analysis.

### 2.2. MBD Cap-Seq Analysis

Methylated DNA was obtained by the MethylMiner Methylated DNA Enrichment Kit (Invitrogen, Carlsbad, CA, USA) according to the manufacturer’s instructions. Briefly, one microgram of genomic DNA was sonicated and captured by MBD proteins. The methylated DNA was eluted in 1 M salt buffer. DNA in each eluted fraction was precipitated by glycogen, sodium acetate and ethanol, and resuspended in TE buffer. Eluted DNA was used to generate libraries following the standard protocols from Illumina. Next, MBD Cap-seq libraries were sequenced using the Illumina Genome Analyzer II as per manufacturer’s instructions. Image analysis and base calling were performed with the standard Illumina pipeline.

Single-end 50 bp reads were mapped to the University of California Santa Cruz (UCSC) rat transcriptome (rn5) by Bowtie with parameters-v 2-best-k 10. Multiple matched reads were processed by LONUT [34], an algorithm developed originally to improve the detection of the enriched regions for ChIP-seq and MBD-seq data. The multiple matched reads close to peaks detected using the uniquely matched reads were retained and combined with uniquely matched reads. The combined reads were then binned by 100 bp bin-size and normalized by total reads of each sample.

Each transcript in UCSC RefSeq database with a unique transcription start site (TSS) and termination site (TES) was further divided into four genomic regions as described in Supplementary Figure S2. Distal (upstream 2–100 kb), TSS (upstream and downstream 2 kb of 5’), TES (upstream and downstream 2 kb of 3’), and Genic or gene body region (down 2 kb of 5’ to up 2 kb of 3’). The reads within these four genomic regions were then used for the differential methylation analysis. Differential methylation level at each bin (100 bp) was determined by a Wilcoxon rank-sum test between each exposure group and control group. For TSS, TES and Distal regions, a minimum of 2 consecutive bins with a *p*-value less than 0.05, a minimum of 0.2 rpm on average in higher methylated group, and a minimum log2FC of 1 was considered for a statistically significant differential methylation region (DMR).

### 2.3. Bioinformatics Analysis

Pathway Analysis: Genes showing inverse correlation in methylation and expression data were selected for analysis using Ingenuity pathway analysis. The expression fold change values were used to score the genes from prepubertal BPA, GEN and BPA + GEN exposure groups to determine the enriched canonical pathways and networks in each list.

Survival analysis: The overall patient survival time was obtained for each patient in TCGA cohort [35]. A total of 738 patients were categorized into 567 estrogen receptor α+ (ERα+) and 171 ERα− patient groups. The patient expression data was compared with control tissue samples to identify patients showing up-regulated and down-regulated expressions. Up- and down-regulated values were converted to 1 and 0 binary codes to do Kaplan-Meier survival and Cox proportional hazard model analysis. For boxplots, statistical significance was assigned as, ** if *p* < 0.01, and *** if *p* < 0.001.

## 3. Results

### 3.1. Identification of Differentially Methylated Loci Following Prepubertal Exposure to BPA and/or GEN in Rats

**Figure 1:**
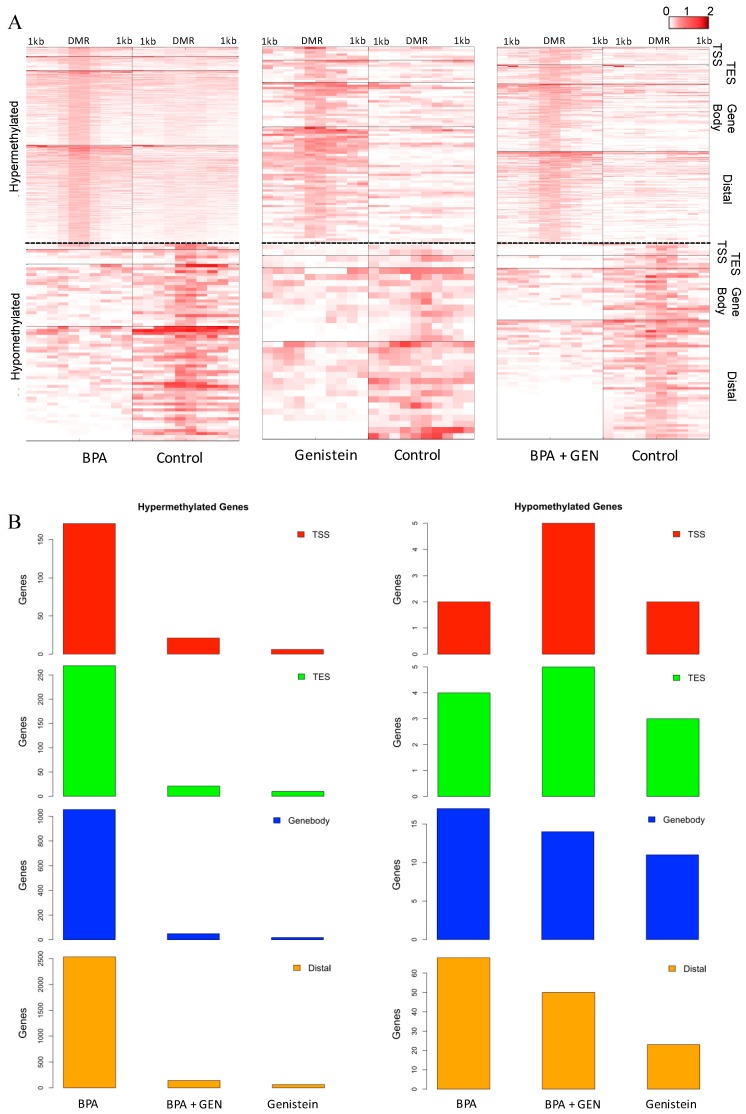
DNA methylation profiles of distal, transcription start site (TSS), termination site (TES), and genebody regions of genes in adult mammary glands of rats exposed prepubertally to Bisphenol A (BPA) ± genistein (GEN). Methyl capture sequencing (MBDCap-seq) was used to generate DNA methylation profiles of the distal, TSS, TES, and genebody regions of genes in mammary glands of 100 day old rats exposed prepubertally to BPA, GEN, BPA + GEN, and Controls. (**A**) The heat maps show hyper- (above the line) and hypo- (below the line) methylated genes in BPA (*n* = 4119), GEN (*n* = 134), and BPA + GEN (*n* = 309) groups; (**B**) Bar chart shows total number of hypo/hypermethylated genes for each region.

To explore how long-term DNA methylation patterns are affected following prepubertal exposure to BPA or GEN, we conducted MBDCap-seq [36] on mammary glands procured from 100 day-old rats exposed prepubertally to vehicle control, BPA, GEN, or BPA + GEN (*n* = 5/group) via the lactating dam. We sequenced ≈800 million raw and ~500 million unique-mapped reads for all samples (Supplementary Figure S1). After applying LONUT [37], a program developed to recover unmapped or multiple-mapped reads, and the total of mapped reads useful for further data analysis were increased by 20–30%. We subsequently examined loci near the TSS representing the promoter regions along with distal, gene-body and near the TES regions (Supplementary Figure S2) for ≈25,000 RefSeq annotated genes [30,38]. After applying Wilcoxon rank-sum test to compare two consecutive 100-bp bins within each of four sub-regions between BPA ± GEN exposed vs. control groups, we found that a large number of gene loci were differentially methylated in these sub-regions. In total, using a cutoff of *p*-value less than 0.05 and 2-fold change, we identified 4, 119, 134, and 305 genes with differentially methylated loci in mammary glands of 100 day-old rats exposed prepubertally to BPA, GEN, or BPA + GEN, respectively, compared to controls (Figure 1A & Supplementary Table S1). We further characterized the regions according to hypo/hypermethylation status observed in each exposure group (Figure 1B). We identified large number of loci hypermethylated in BPA whereas the number of loci hypermethylated in GEN and BPA + GEN were considerably low (Supplementary Figure S3).

### 3.2. Network and Pathway Analyses of DNA Methylated Targets Mediated by Prepubertal BPA and/or GEN Exposures

**Figure 2:**
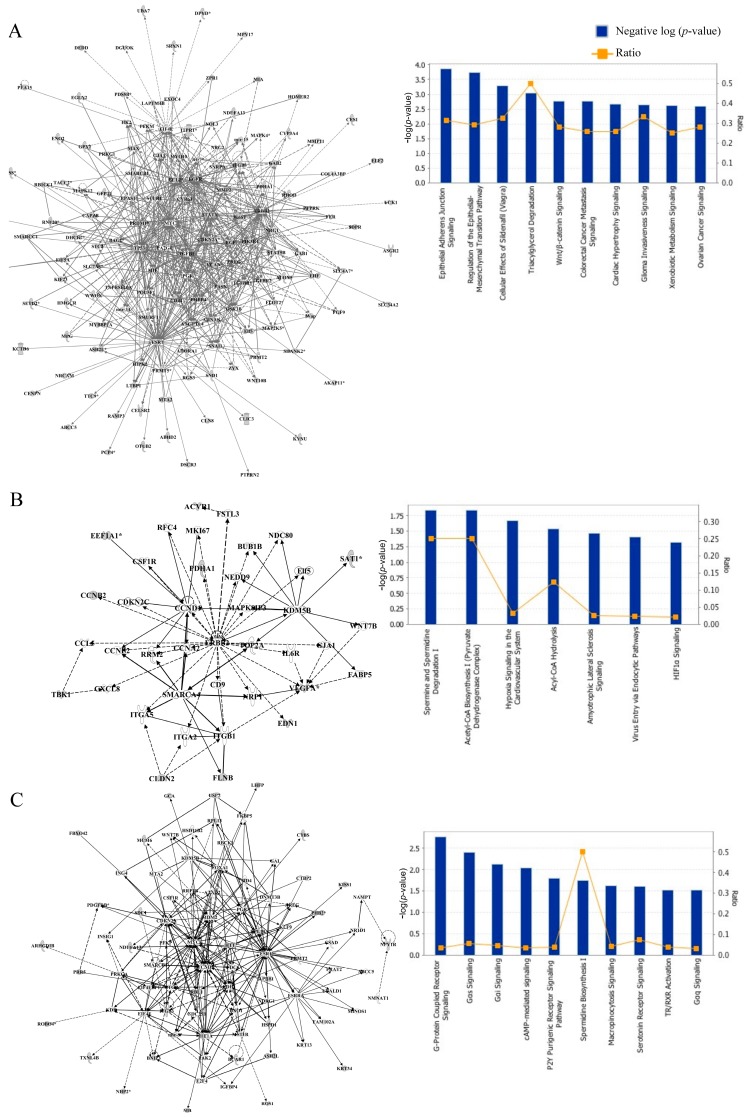
Network and pathway analyses of hypermethylated genes in adult mammary glands of rats exposed prepubertally to BPA ± GEN. Differentially methylated genes were used to predict interaction networks and pathways that could be dysregulated. Interaction network and pathway analyses were carried out for exposure groups: (**A**) BPA; (**B**) GEN; (**C**) Combined BPA + GEN. The bar plot illustrates the level of gene enrichment present in the listed pathways.

To understand how differentially methylated genes function in the mammary glands of animals prepubertally exposed to BPA, GEN, and BPA + GEN, we carried out network and pathway analyses using QIAGEN’s Ingenuity Pathway Analysis (Supplementary Table S2). Top interaction networks following prepubertal BPA exposure were predominantly related to cellular assembly and organization, cellular compromise, cellular development, cancer, organismal injury and abnormalities, reproductive system disease, cell death and survival, cellular movement, cell cycle, and cell morphology (Figure 2A & Supplementary Figure S4A). On the other hand, genes differentially methylated following GEN exposure were associated with cellular assembly and organization, cellular compromise, and post-translational modification (Figure 2B & Supplementary Figure S4B). Top interaction networks for combinational exposure to BPA + GEN identified genes associated with cellular assembly and organization, molecular transport, nucleic acid metabolism, and cellular function and maintenance (Figure 2C & Supplementary Figure S4C).

### 3.3. In Silico Correlation of Rat DNA Methylated Genes with Breast Cancer Patient Data from the Cancer Genome Atlas (TCGA)

**Figure 3:**
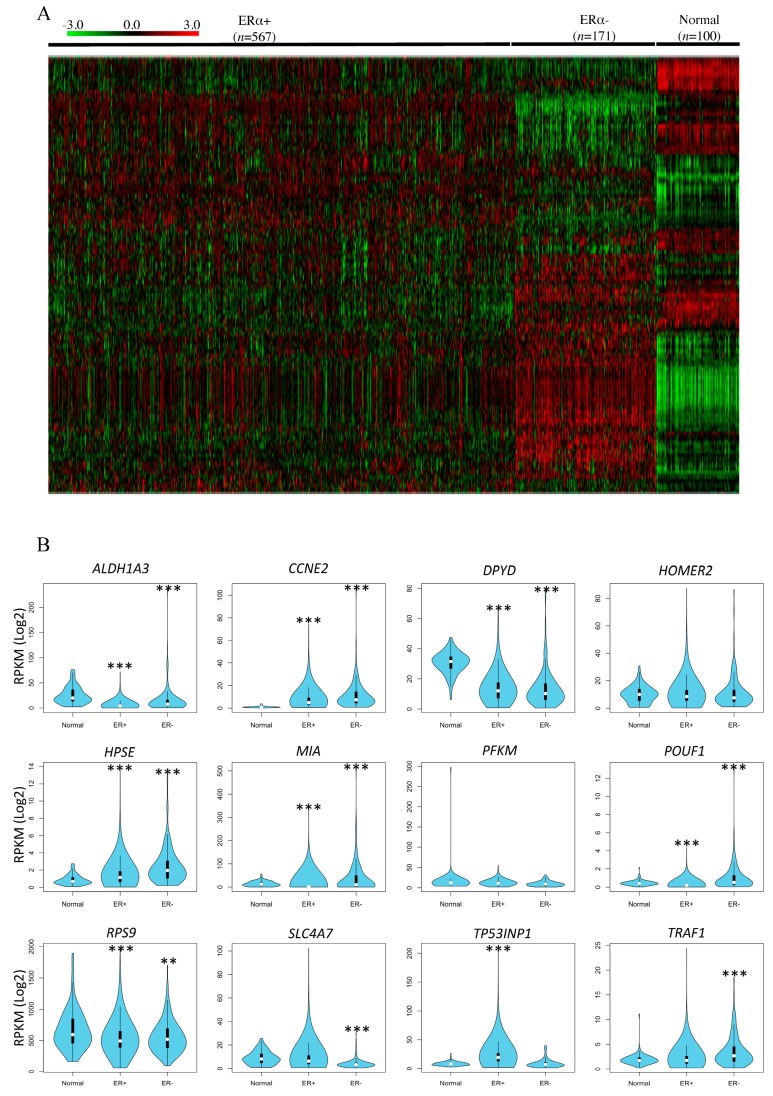
In silico correlation of rat mammary methylated genes with breast cancer patient data from The Cancer Genome Atlas (TCGA). (**A**) A heat map of the TCGA breast cancer patient data showing gene expression patterns in the 155 selected genes across estrogen receptor α+ (ERα+), ERα- patients and normal samples. The −3 to +3 bar is the gradient for the colors in the heat map indicating low to high expression, respectively; (**B**) The box plots show differences in average gene expression in each ER subsets compared to normal tissue as described in Methods section.

Numerous studies have demonstrated the contrasting roles of BPA and GEN in mammary cancer susceptibility [2,3,11,33,34,35,39,40,41]. Hence, we determined whether the differentially methylated genes identified in the mammary glands of rats exposed prepubertally to BPA, GEN, or BPA + GEN are also differentially regulated at the gene level in breast cancer patients. Using the genes identified in rats that are differentially methylated by xenoestrogen exposure, we investigated their gene expression in the TCGA breast cancer cohort [42] (Supplementary Figure S5). Since BPA and GEN have been associated with estrogen action, we stratified patients into ERα+ and ERα− groups [43]. We found a large difference in the expression levels of many of these genes in the tumors of the ERα+ and ERα− patients as compared to normal tissue samples (Figure 3A & Supplementary Figure S6).

We investigated 291 genes that were identified from top networks associated with each exposure group. Out of these, there were 155 genes with significant differences in their gene expressions compared to normal tissue samples. As BPA and GEN are associated with estrogen action, we selected the genes which showed significant differences in ERα+ patient groups and were also differentially methylated.

### 3.4. Hazard Ratio and Survival Analysis of rat DNA Methylation Regulated Targeted Genes in Breast Cancer Patients

**Figure 4:**
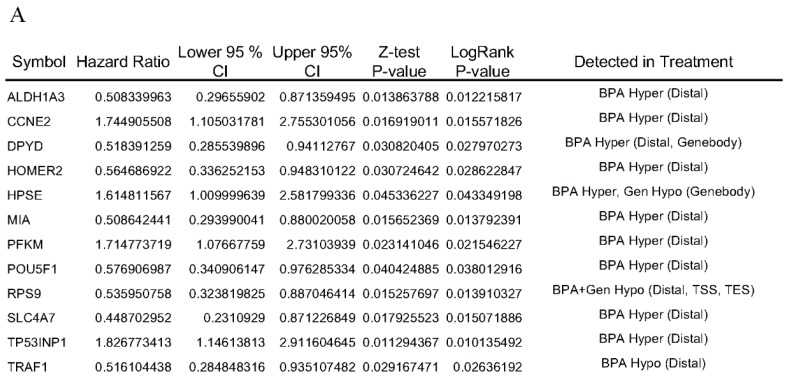

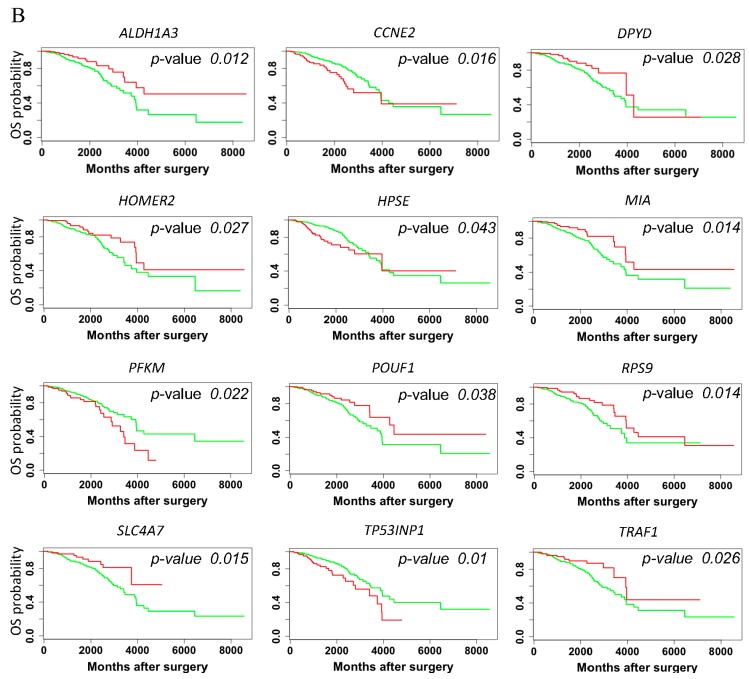
Hazard ratio and survival analysis of rat methylated genes with human breast cancer patient data from the TCGA network. (**A**) Expression values measured by RNA-seq data were assessed with survival time for breast cancer cohort patients; (**B**) Kaplan-Meier curves for the rat mammary gland genes identified to have potential to predict survival via the ERα+ and ERα− patient data of TCGA network. The green lines are the ERα+ patients showing lower gene expression than the top 75 percentile of patients and the red are the ERα+ patients showing higher gene expression.

Next, we investigated these 291 genes for prognostic values on patient survival in ERα+ patient groups. We found twelve of these genes showed significant association with survival along with altered DNA methylation profiles. Box plots in Figure 3B illustrate most of these 12 genes identified with the human data sets also exhibit significant differences in patients and in our exposure groups. Four genes identified as having strong predictive values for poor survival in ERα+ patients via log-rank test and hazard ratios were *CCNE2*, *HPSE*, *PFKM*, and *TP53INP1* (Figure 4A,B). Higher expression of these genes corresponded to higher risk and shorter term survival in patients. In contrast, eight genes with predictive values for long term patient survival in ERα+ patients were *ALDH1A3*, *DPYD*, *HOMER2*, *MIA*, *POU5F1*, *RPS9*, *SLC4A7*, and *TRAF1*. Nine of these 12 genes were identified as being hypermethylated in distal region for the rat BPA exposure group (Figure 4A). RPS9 was hypo-methylated in BPA + GEN exposure group in distal, TSS and TES regions. Also, TRAF1 was hypo-methylated in BPA exposure group in distal region. Importantly, HPSE was hypermethylated in BPA and hypomethylated in GEN both in the gene body region and indicated patients with higher expression having poor overall survival.

## 4. Discussion

Despite studies showing that early postnatal exposure to hormonally-active chemicals are capable of exerting long-lasting biochemical changes at the cellular level and to the proteome, and for susceptibility for cancer [2,3,11,33,34,35,39,40,41,44], there is scant evidence on the underlying mechanism of how and to what extent this occurs. Accordingly, it was our goal to investigate DNA methylation as a mechanism of altering epigenetic effects and to determine if this can be applied to a clinical situation. For this, we chose two chemicals that have been extensively studied for their potential to predispose for and protect against chemically induced mammary cancer. The exposure protocol is unique for teasing this out. By treating the lactating dam shortly after birth, the nursing offspring are not exposed to undue stress, and at the end of the exposure period—at time of weaning (day 21 postnatally)—the chemical exposure diminishes via metabolism and disposition to the point that the effecting chemical(s) are no longer around to directly influence outcomes at postnatal day 100 [11,34]. Using this protocol, we can also determine if GEN can counter or negate the effect of BPA on gene expression by assessing DNA methylation after exposure occurs simultaneously during a critical period of postnatal development, i.e., prepubertally in rats. Finally, after evaluating the potential of these epigenetic changes to alter cancer susceptibility in the rat model, we assessed the potential of these epigenetic changes in TCGA patient tissues.

BPA and GEN have previously been shown to directly affect the expression of DNA methytransferases (DNMTs) and other genes [28,45,46,47], however our study was not designed to look at the short-term direct effect, but rather the long-term effects following early exposure only (days 2–21 postnatally) to these two chemicals. Our data shows that there are substantial effects even in the absence of the original effectors resulting in significantly different phenotypes in the mammary tissue of adult rats. To further characterize the differentially methylated genes, we performed network and pathway analysis for genes in each exposure group. As there were a large number of differentially methylated genes in the BPA exposure group, we identified many enriched networks, most of them related to cancer, cell death and survival, cell movement, cellular growth and proliferation. Interestingly, although very few genes overlapped between the three exposure groups (Supplementary Figure S5), genes in GEN and BPA + GEN exposure groups were enriched in distinct networks than the ones identified in the BPA exposure group. This can be an indication of alternative mechanisms by GEN for its chemopreventive role to counter the effects of BPA.

We further combined the differentially methylated genes and those predicted in networks for each exposure group to investigate their gene expressions. Even though our data were obtained from rat mammary glands, we identified altered gene expressions in breast cancer patient data derived from TCGA for our target genes. We found that many of those genes were differentially expressed in either ERα+ patients or ERα− patients in comparison to normal tissues (Figure 3A). We identified 12 genes with potential prognostic value in patients which were also found to be differentially methylated in rat exposure groups.

The four genes identified as having strong predictive values for poor survival in TCGA breast cancer cohort, *CCNE2*, *HPSE*, *PFKM*, and *TP53INP1* are especially intriguing because of their potential importance for cancer causation or prevention, survival, and as biomarkers of susceptibility. Looking at the biological functions of CCNE2, HPSE, and PFKM proteins, they are for the most part, involved in cell cycle G1/S transition, proliferation and carcinogenesis, and associated with breast cancer [48,49,50]. On the other hand, TP53INP1 has been associated with cell growth arrest and apoptosis by modulating p53 transcription [51], i.e., it acts as a tumor suppressor. The rest of the genes showed high expression and low hazardous ratio (longer survival prediction) in ERα+ TCGA patients. Of particular interest is HPSE. In the mammary glands of adult rats exposed prepubertally to GEN, *HPSE* was found to be hypomethylated in the gene body region, whereas it was hypermethylated in the same region of mammary glands of prepubertal BPA–exposed animals. In accordance with recent studies showing gene body methylation to be positively correlated to gene expression [52], hypomethylation of HPSE could result in downregulation in GEN exposed animals, whereas hypermethylation in BPA exposed rats could result in upregulation of the gene product, opposing effects in two chemicals have been shown to have opposing predisposition for carcinogenesis [2,10,11,18,19,20,33].

While prepubertal BPA exposure resulted in many genes being hypermethylated, prepubertal GEN and prepubertal combinational GEN + BPA exposures resulted in few hypermethylations. This would suggest that GEN is less active as a methylation agent than BPA, but as reported by Wang et al., prepubertal GEN does oppose the effects of prepubertal BPA exposure on cell proliferation and apoptosis in the mammary gland of adult rats [3,33]. In fact, GEN has been shown to cause many post-transcriptional modifications (phosphorylations, glycosylations, and acetylations) to proteins regulating cell cycle regulation and apoptosis, resulting in protecting against toxicity, including carcinogenesis [3,33,34,53,54]. These selective modifications are consistent with the prepubertal BPA exposed rats being more susceptible for mammary cancer, and prepubertal GEN exposed rats being less susceptible for chemically-induced mammary cancer [2,10,11,18,19,20,21,22,33].

Overall, our analyses reveal that short-term postnatal exposures to the hormonally-active and toxic compound BPA result in a large number of long-term epigenetic modifications in the mammary tissue. As these are modifications in DNA methylation patterns, they remain stable for a long time and could be used as potential pre-diagnostic epigenetic markers indicating the resulting effects of these compounds. Furthermore, the genes and pathways identified as being altered in these exposure groups reinforces the potential carcinogenic effect of BPA and chemo-preventive effect of GEN. The combined treatment of BPA and GEN revealed networks similar to those enriched in BPA exposure, indicating that the presence of GEN either reduces or counters the alterations caused by the carcinogenic properties of BPA alone by altering alternate genes in the same networks. Investigating the expression of these altered genes from the exposure groups in actual breast cancer patients enabled us to identify potential prognostic markers. Furthermore, four of these genes –*CCNE2*, *HPSE*, *PFKM*, and *TP53INP1*—are shown to predict poor long term survival in ERα+ TCGA patients. Genistein appears to act primarily via post translational modifications.

## Figures and Tables

**Table 1:** Animal Treatments.

Group Identification	Gavage Administered	Food Administered
(1) Control (SO)	Sesame Oil as Vehicle	AIN-76A
(2) Bisphenol A (BPA)	250 µg BPA/kg BW	AIN-76A
(3) Genistein (GEN)	Sesame Oil as Vehicle	250 mg genistein/kg AIN-76A diet
(4) BPA + GEN	250 µg BPA/kg BW	250 mg genistein/kg AIN-76A diet

## Supplementary Materials

The following are available online at www.mdpi.com/2073-4425/8/5/144/s1. Supplementary Figure S1: Figure showing the total number of reads obtained from sequencing each sample (Fastq), reads uniquely mapped to the genome (UMT), reads not uniquely mapped to the genome (NUT), and total recovered reads using LONUT (CMT). Supplementary Figure S2: Illustration of the investigated genomic regions and how these different regions defined in the DNA methylation analysis were selected. Supplementary Figure S3: Overlap of hyper and hypomethylated genes identified from each exposure group. Supplementary Figure S4: Network and pathway analyses of hypomethylated genes in adult mammary glands of rats exposed prepubertally to BPA ± GEN. Supplementary Figure S5: Overlap of differentially methylated genes along with the genes interacting with them as identified in top 5 networks for each exposure group. Supplementary Figure S6: Boxplots for genes identified to be differentially expressed in TCGA patient groups compared to normal. Supplementary Table S1: List of genes identified to be differentially methylated in each exposure group compared to controls. Supplementary Table S2: Top 5 networks identified in each exposure group along with the list of genes belonging to them.

## Data Access

Raw and processed MBD-seq data and gene expression data for 20 Rats are being deposited in GEO under accession number GSE89107 (http://www.ncbi.nlm.nih.gov/geo/).
