# Supplementary material for: DNA Methylation Targets Influenced by Bisphenol A and/or Genistein Are Associated with Survival Outcomes in Breast Cancer Patients
Source: Genes (Basel). 2017 May 15;8(5):144. doi: 10.3390/genes8050144 (PMC5448018; doi:10.3390/genes8050144)
Supplement: Supplementary file 1 [file genes-08-00144-s001.zip › genes-186435-supplementary.pdf]

DNA Methylation Targets Influenced by Bisphenol A and/or Genistein Are Associated with Survival Outcomes in Breast Cancer Patients

Rohit R. Jadhav, Julia Santucci-Pereira, Yao V. Wang, Joseph Liu, Theresa D Nguyen, Jun Wang, Sarah Jenkins, Jose Russo, Tim H.-M. Huang, Victor X. Jin and Coral A. Lamartiniere

Supplementary Figure S1

Samples	Fastq	UMT	Recovered NUT	CMT
Sample_BPA_3029	40.8	27.7	10.7	38.4
Sample_BPA_3030	40.5	27.4	11	38.4
Sample_BPA_3031	39.1	26.9	10.2	37.1
Sample_BPA_3032	37.8	25	10.8	35.8
Sample_BPA_3033	39.9	26.1	11.6	37.6
Sample_BPA_Gen_3330	39.5	27.9	9.7	37.7
Sample_BPA_Gen_3331	35.9	24.7	9.4	34.1
Sample_BPA_Gen_3332	44.4	30.3	11.8	42.1
Sample_BPA_Gen_3333	38.2	24.9	11.1	36
Sample_BPA_Gen_3334	45.6	29.3	13.7	43
Sample_CTRL_3229	41.4	26.1	13	39
Sample_Ctrl_3232	44.9	30.9	11.2	42.1
Sample_Ctrl_3233	37.9	23.9	11.9	35.8
Sample_Ctrl_3235	44.8	31	11.1	42.1
Sample_Ctrl_3236	33.5	20.6	11	31.6
Sample_Genistein_3280	53.6	32.2	18.2	50.4
Sample_Genistein_3281	44.6	27.5	14.7	42.2
Sample_Genistein_3282	46.7	31.1	12.9	44
Sample_Genistein_3283	58.4	39.4	15.7	55.1
Sample_Genistein_3284	36.4	25.3	8.9	34.2

Supplementary Figure S2

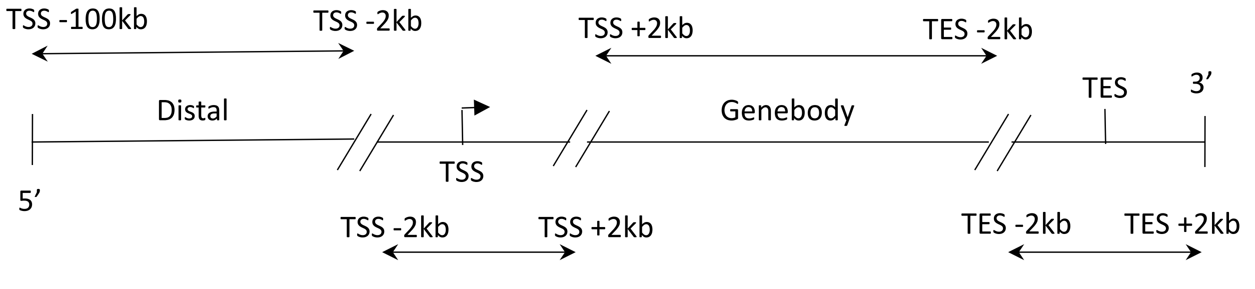

Supplementary Figure S3

Hyper-methylated Genes

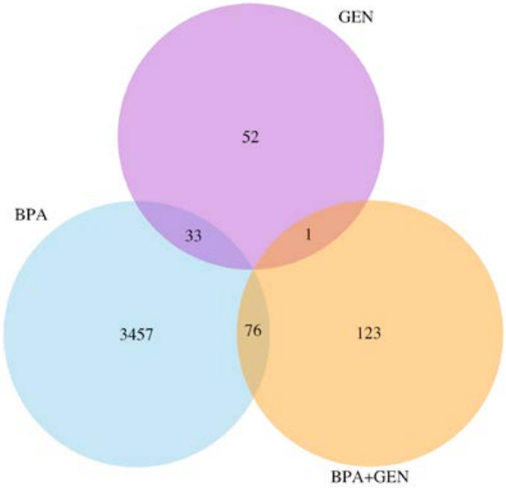

Hypo-methylated Genes

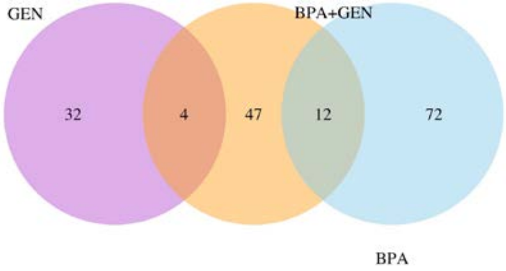

Supplementary Figure S4

A

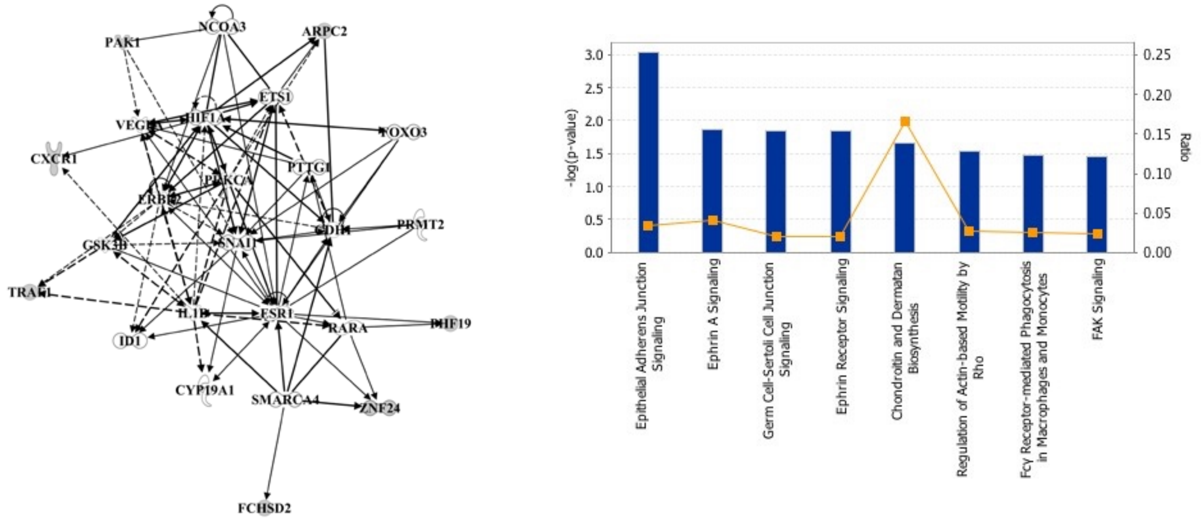

B

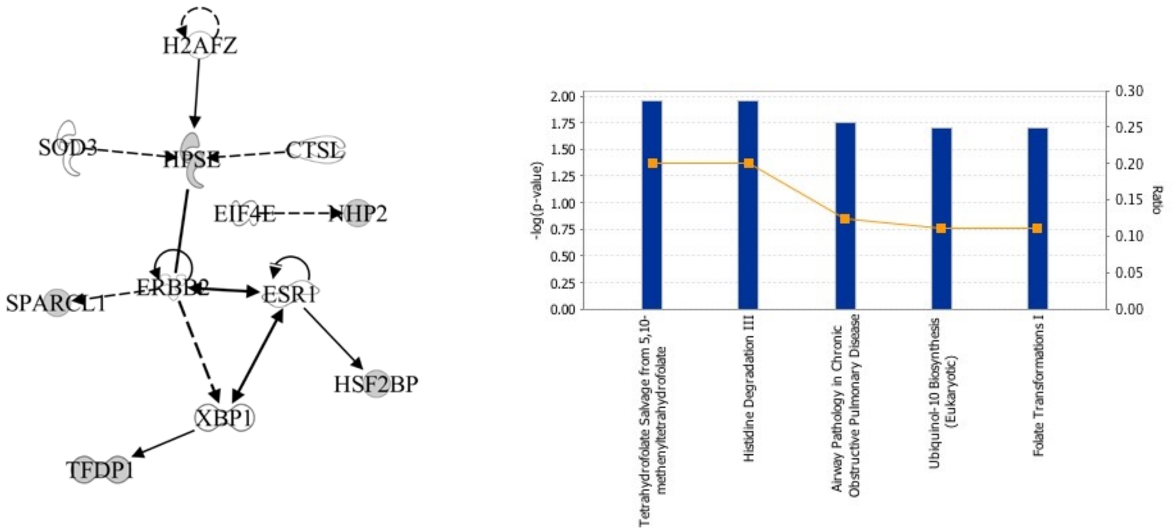

C

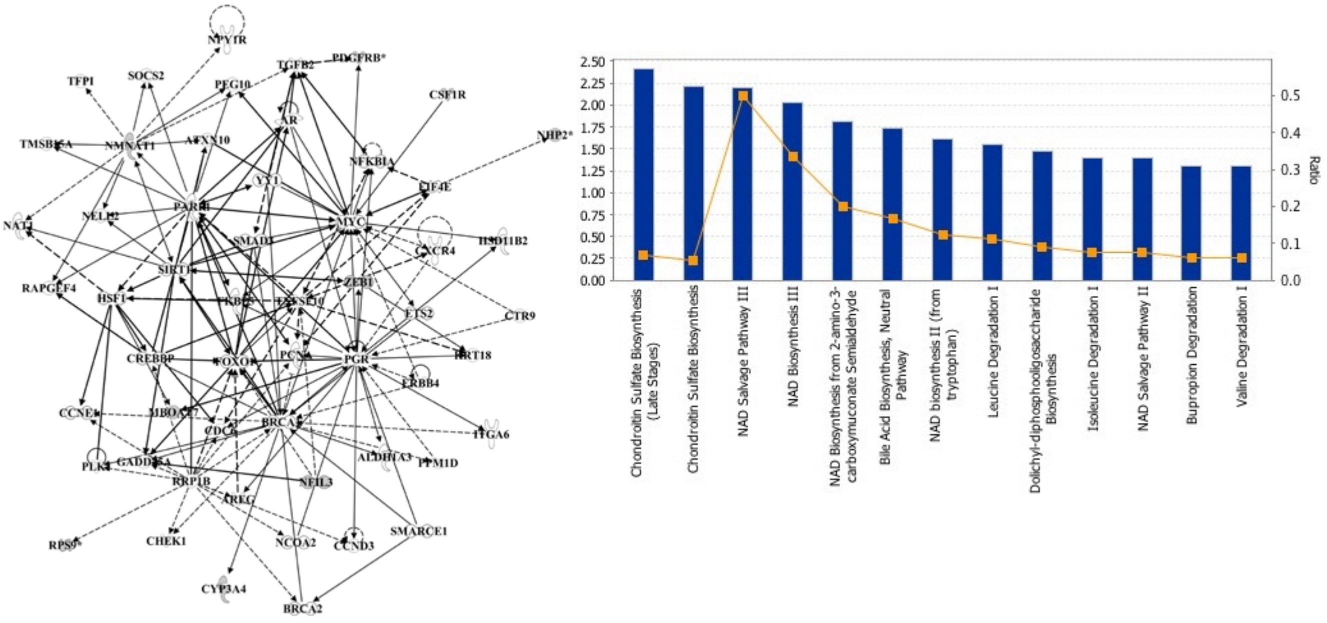

Supplementary Figure S5

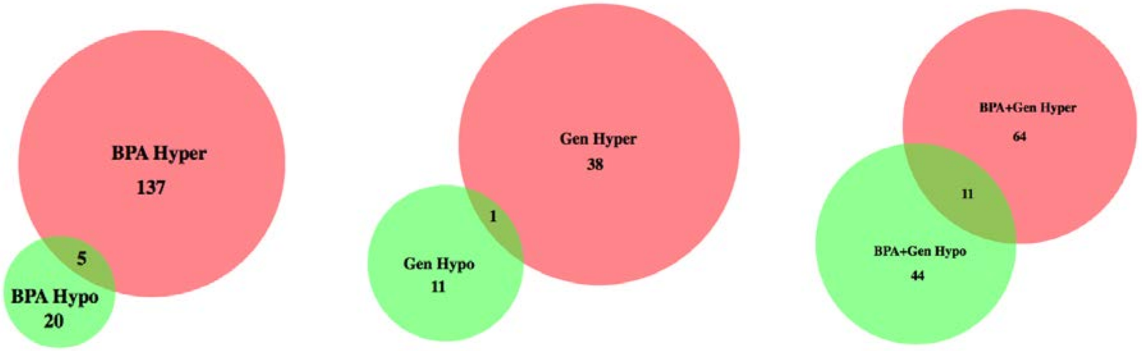

Supplementary Figure S6

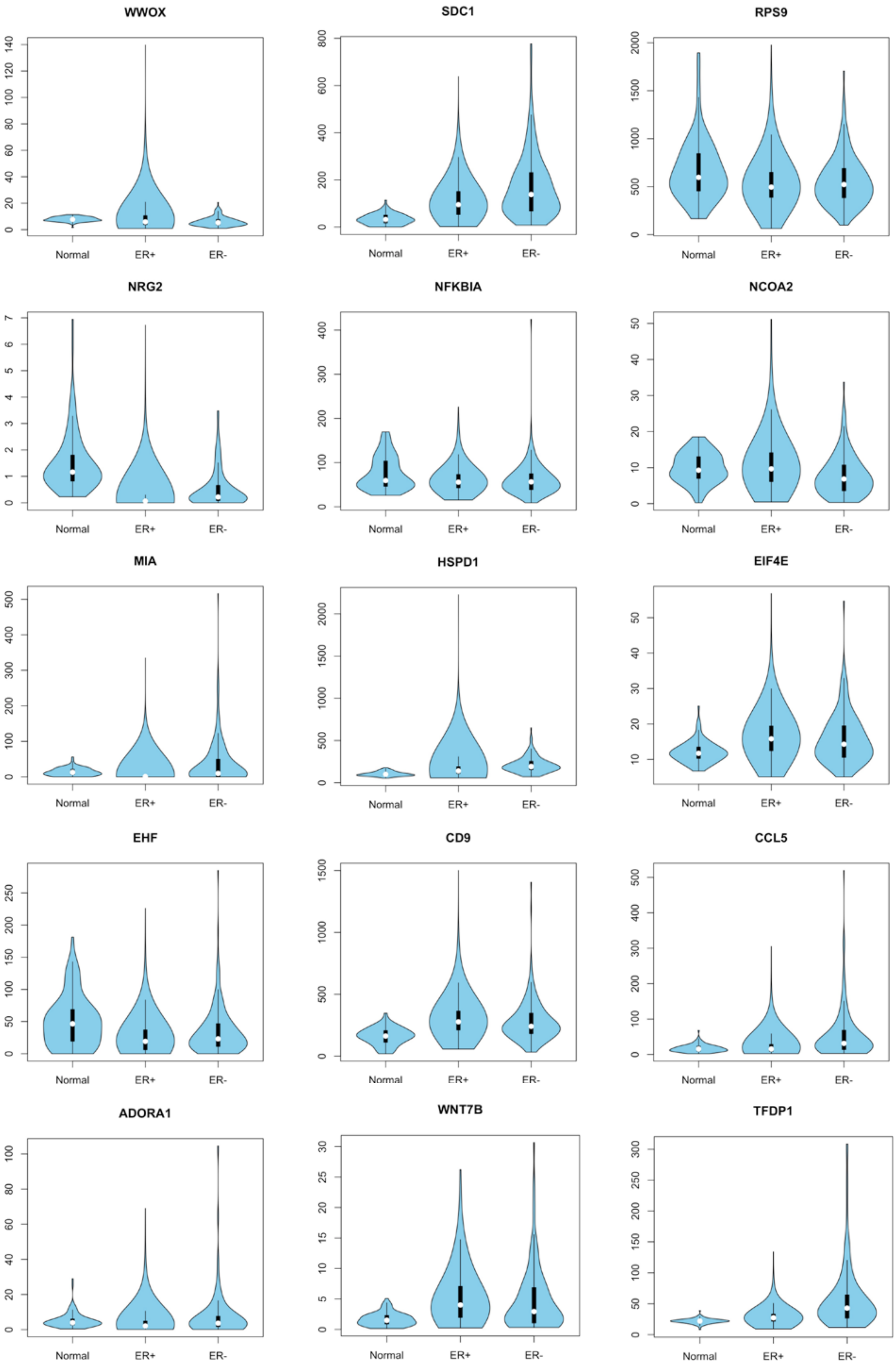

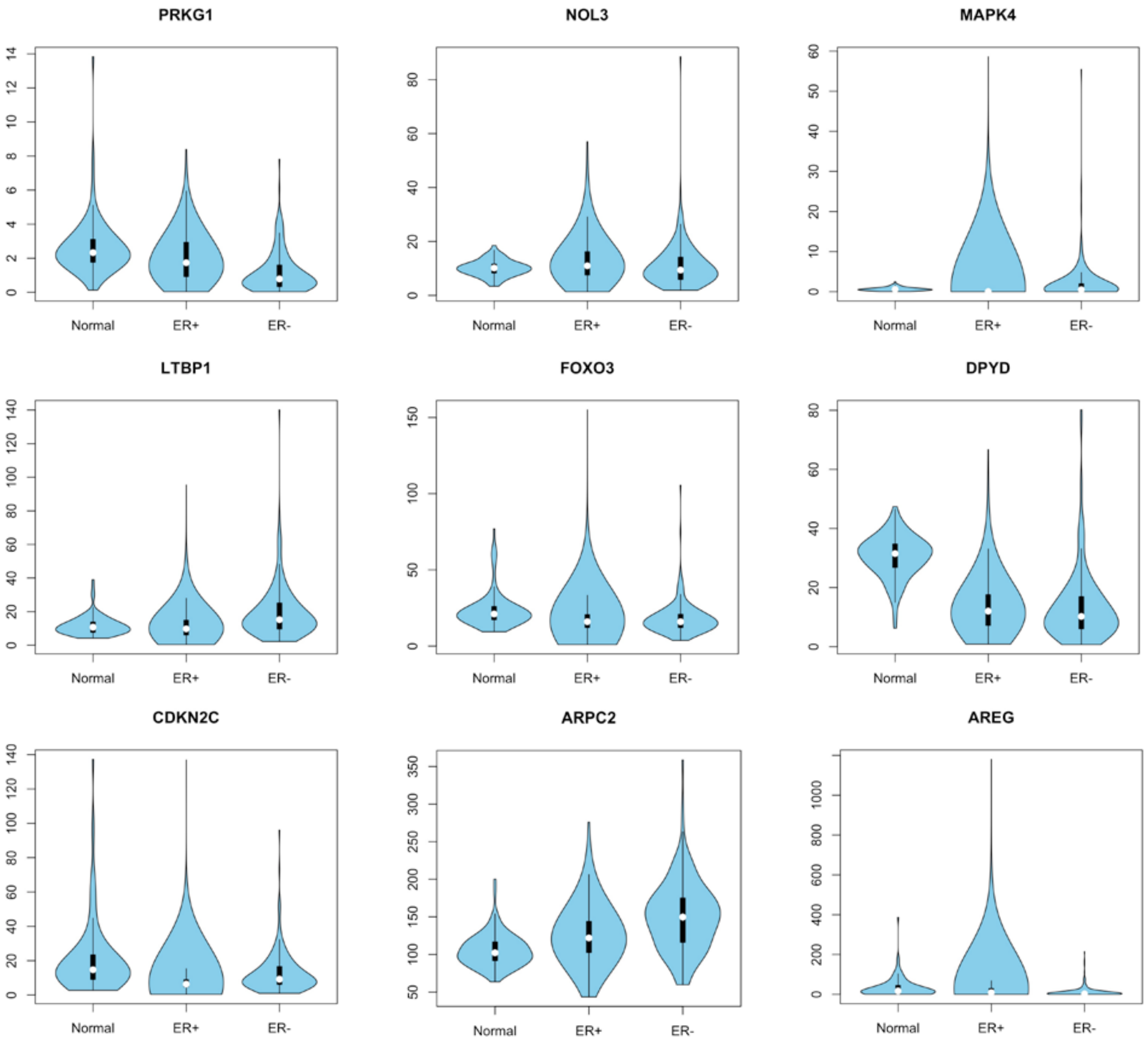
PRKG1
NOL3
MAPK4
LTBP1
FOXO3
DPYD
CDKN2C
ARPC2
AREG
Normal
ER+
ER-

Supplementary Figures

Supplementary Fig. S1: Figure showing a total number of reads obtained from sequencing each sample (Fastq), reads uniquely mapped to the genome (UMT), reads not uniquely mapped to the genome (NUT), and total recovered reads using LONUT (CMT).
Supplementary Fig. S2. Illustration of the investigated genomic regions and how these different regions defined in the DNA methylation analysis were selected.
Supplementary Fig. S3. Overlap of hyper and hypo methylated genes identified from each exposure group.
Supplementary Fig. S4. Network and pathway analyses of hypomethylated genes in adult mammary glands of rats exposed prepubertally to BPA ± GEN.
Supplementary Fig. S5. Overlap of differentially methylated genes along with the genes interacting with them as identified in top 5 networks for each exposure group.
Supplementary Figure S6. Boxplots for genes identified to be differentially expressed in TCGA patient groups compared to normal.

Supplementary Tables

Supplementary Table S1: List of genes identified to be differentially methylated in each exposure group compared to controls.
Supplementary Table S2: Top 5 networks identified in each exposure group along with the list of genes belonging to them.
